# Physics of cell adhesion: some lessons from cell-mimetic systems

**DOI:** 10.1039/c3sm51910d

**Published:** 2014-03-21

**Authors:** Erich Sackmann, Ana-Sunčana Smith

**Affiliations:** a Physics Department Technical University Munich, Germany; b Department of Physics, Ludwig-Maximillian University, Munich, Germany; c Institute for Theoretical Physics, Friedrich Alexander University Erlangen-Nürnberg, Erlangen, Germany; d Institute Rud̷er Bošković, Zagreb, Croatia. smith@physik.fau.de

## Abstract

Cell adhesion is a paradigm of the ubiquitous interplay of cell signalling, modulation of material properties and biological functions of cells. It is controlled by competition of short range attractive forces, medium range repellant forces and the elastic stresses associated with local and global deformation of the composite cell envelopes. We review the basic physical rules governing the physics of cell adhesion learned by studying cell-mimetic systems and demonstrate the importance of these rules in the context of cellular systems. We review how adhesion induced micro-domains couple to the intracellular actin and microtubule networks allowing cells to generate strong forces with a minimum of attractive cell adhesion molecules (CAMs) and to manipulate other cells through filopodia over micrometer distances. The adhesion strength can be adapted to external force fluctuations within seconds by varying the density of attractive and repellant CAMs through exocytosis and endocytosis or protease-mediated dismantling of the CAM–cytoskeleton link. Adhesion domains form local end global biochemical reaction centres enabling the control of enzymes. Actin–microtubule crosstalk at adhesion foci facilitates the mechanical stabilization of polarized cell shapes. Axon growth in tissue is guided by attractive and repulsive clues controlled by antagonistic signalling pathways.

## Introduction

The main purpose of this review is to show that model membrane studies can provide insights into the physical basis of cell recognition and adhesion processes in several ways: (i) these studies teach us how to quantify adhesion by measuring free adhesion energies, Δ*G*_ad_, or unbinding forces. (ii) Mimetic models show that the formation of adhesion domains is an inevitable consequence of the interplay of short range attraction forces between pairs of cell adhesion molecules (CAMs), long range repulsion forces mediated by repeller molecules of the glycocalyx, and elastic stresses of the lipid protein bilayer. (iii) Reconstituted membranes reveal a fundamental difference between cell–cell adhesion with all CAMs mobile and cell–matrix interactions with one set of CAMs immobile. (iv) Comparative studies of model systems and cells provide insight into the control of cell adhesion by coupling of the actin heterogels to the adhesion domains, and point to the role of signalling.

Cell-mimetic systems play an important role in gaining insights into the physical basis of cell adhesion and are ideal to study how the mobility, density and topography of CAMs affect the formation of bonds. Furthermore, the effects of elasticity of both the substrate and the cells, which fluctuate and deform in a potential arising from interfacial forces and the glycocalyx can be elucidated. The combination of these elements impacts adhesion energy of microdomains, by affecting the affinity of an isolated CAM complex, but also through cooperative effects between the binding sites, transmitted by the elastic components, as well as through the thermodynamic response of the system.

Many of these effects can be modelled by combining the elasticity theory of adhering elastic shells with the concepts of statistical physics. Consequently, membrane elasticity, the changes of cell shape, and the adhesion energy can be correlated. However, the verification of these theoretical models relies on experiments with model systems containing the essential ingredients of cell adhesion. The first step however involved bringing together the theoretical and experimental approach to develop methods to reliably measure absolute values of adhesion energy. Today, these approaches are used to study the development of the adhesion domains and the above mentioned physical elements which regulate this process.

The adhesion domain formation offers many advantages. Strong cohesive forces between moving cells and their environment can be formed by commitment of a small number (~10^4^) of attractive CAMs. The adhesion strength can be rapidly adapted by varying the density of attractive and repellant CAMs through exocytosis or endocytosis,^1^ and by thermodynamically driven segregation.^2,3^ Furthermore, the strength of the adhesion is affected by changes in the coupling between the adhesion domains and the actin network,^4^ as well as by modulations in the local composition of the membrane, the latter affecting elastic stresses and fluctuations within the domain.^5,6^ Furthermore, adhesion micro-domains, such as immunological synapses, can form biochemical reaction centres which control the access of inhibitors or activators.^7^ Cells can be rapidly polarized by large scale lateral segregation of attractive and repellant CAMs,^8,9^ and by cell shape stabilisation through actin–microtubule cross-talk.^7^ Many elements related to the stated aspects of domain formation occur spontaneously in mimetic systems,^13^ which then provide relevant information for time-scales shorter than those involving the coupling of the intracellular receptor domains to the actin cortex. Since mimetic systems are fully controlled in terms of composition, the physical framework for a given process driving the formation of domains can be established and the role of active processes that follow can be anticipated, which is the general aim of the bottom-up approach.^14,15^

**Fig. 1:**
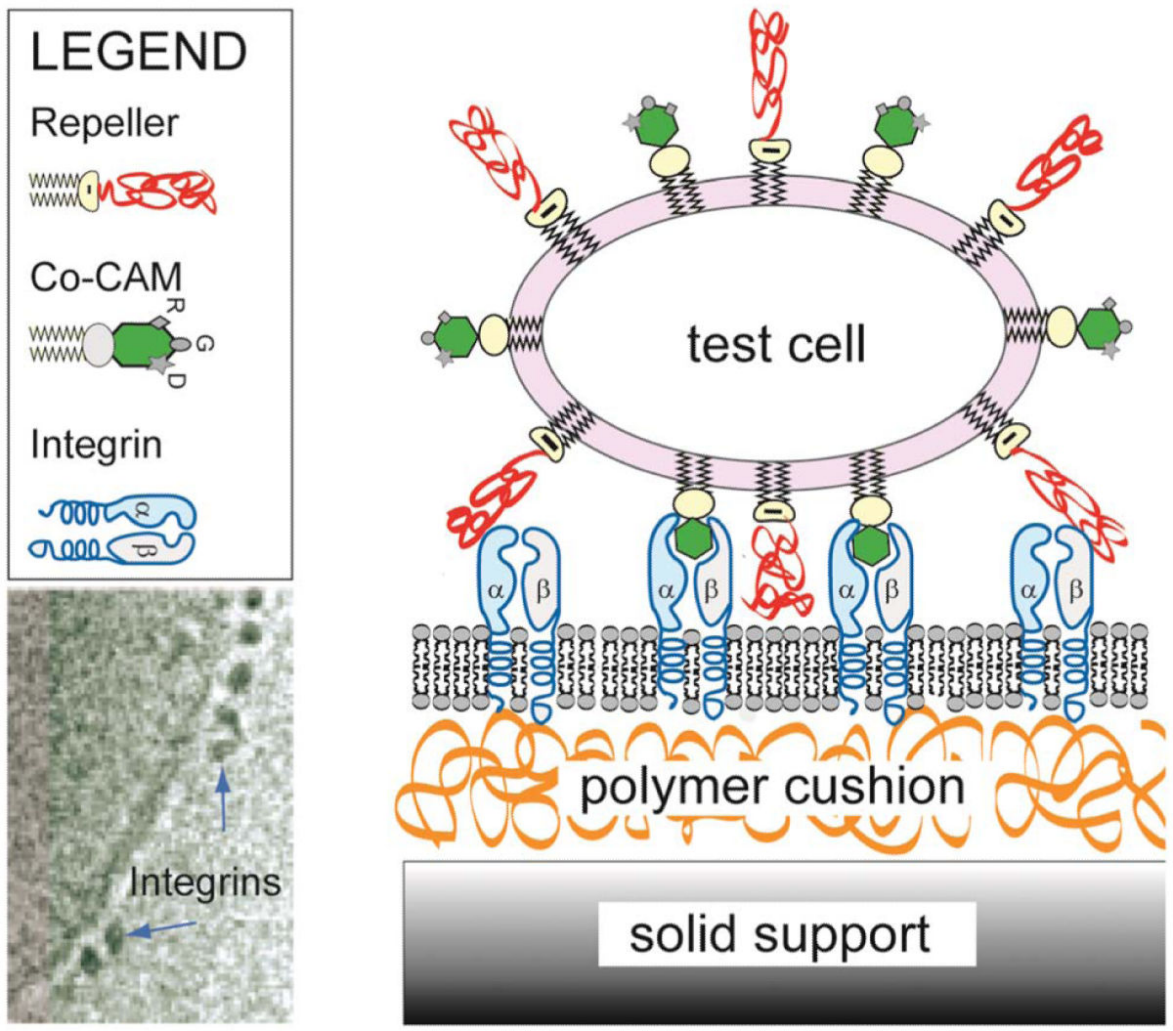
The model system mimicking cell–cell adhesion contains all major ingredients that control the primary process of cell adhesion, which consists of functionalised vesicle binding to a flat substrate acting as a target cell. The latter is generated by fusing vesicles doped with CAM molecules on hydrated polymer cushions, rendering the CAM mobile. In the current example, cyclic peptides exposing RGD sequences in the vesicle specifically recognize freely diffusing integrin α_11_β_3_ on the substrate (diffusivity: *D* ≈ 0.6 μm^2^ s^−1^).^10,11^ The electron microscopy image of reconstituted integrins (bottom left) is reproduced from ref. 12.

In the first part of this review, we describe the essential biomimetic system for domain formation (Fig. 1), and show that pertinent physical parameters (elastic moduli and stresses, tension, free energies of adhesion, unbinding forces, *etc.*) can be measured by analyzing the contour of the soft shells near the surface with reflection interference contrast microscopy (RICM). In the second part, we show that the physical principles learned in biomimetic studies provide new insights into the control of cell–cell and cell–tissue adhesion processes and the modulation of cell material properties controlling adhesion by biochemical signalling. We consider four paradigms of cell adhesion: the stimulation of lymphocyte by encounters with antigen presenting cells through immunological synapses, the penetration of lymphocytes through endothelial cell layers, the global polarisation of cells, and the control of axon pathfinding in tissue by interplay of two counteracting cell signalling pathways.

In summary, studies of cell-mimetic systems are essential to establish relationships between single molecule measurements of CAM–CAM binding forces and the unbinding force of CAMs embedded in membranes. Since the binding energy of membrane bound CAM–CAM pairs is strongly dependent on the thermo-mechanical properties of the composite cell envelope and the actin cortex, the osmotic pressure of unbound CAMs, and generic interfacial forces, these relationships have to be established in order to relate the measurements of single molecule unbinding forces to the binding energy of CAM–CAM pairs embedded in cell membranes.

## Biomimetic models of cell adhesion

Insight into the physical basis of cell adhesion is gained by studying model systems which contain the essential ingredients controlling the primary process of adhesion (Fig. 1), that is before the stabilization of adhesion domains by the actin cortex.^10,11^ In this context, giant vesicles doped with CAMs or ligands of the extracellular matrix (EM) serve as test cells, while solid supported membranes with reconstituted co-receptors or polymer cushion exposing ligands of the EM act as target cells or tissue, respectively.^16^ The role of the glycocalyx is mimicked by doping the membranes with lipids exposing polymer head groups.^17,18^ To mimic the softness of tissue the supported membranes are separated from the solid by ultrathin polymer cushions.^10,11^

**Fig. 2:**
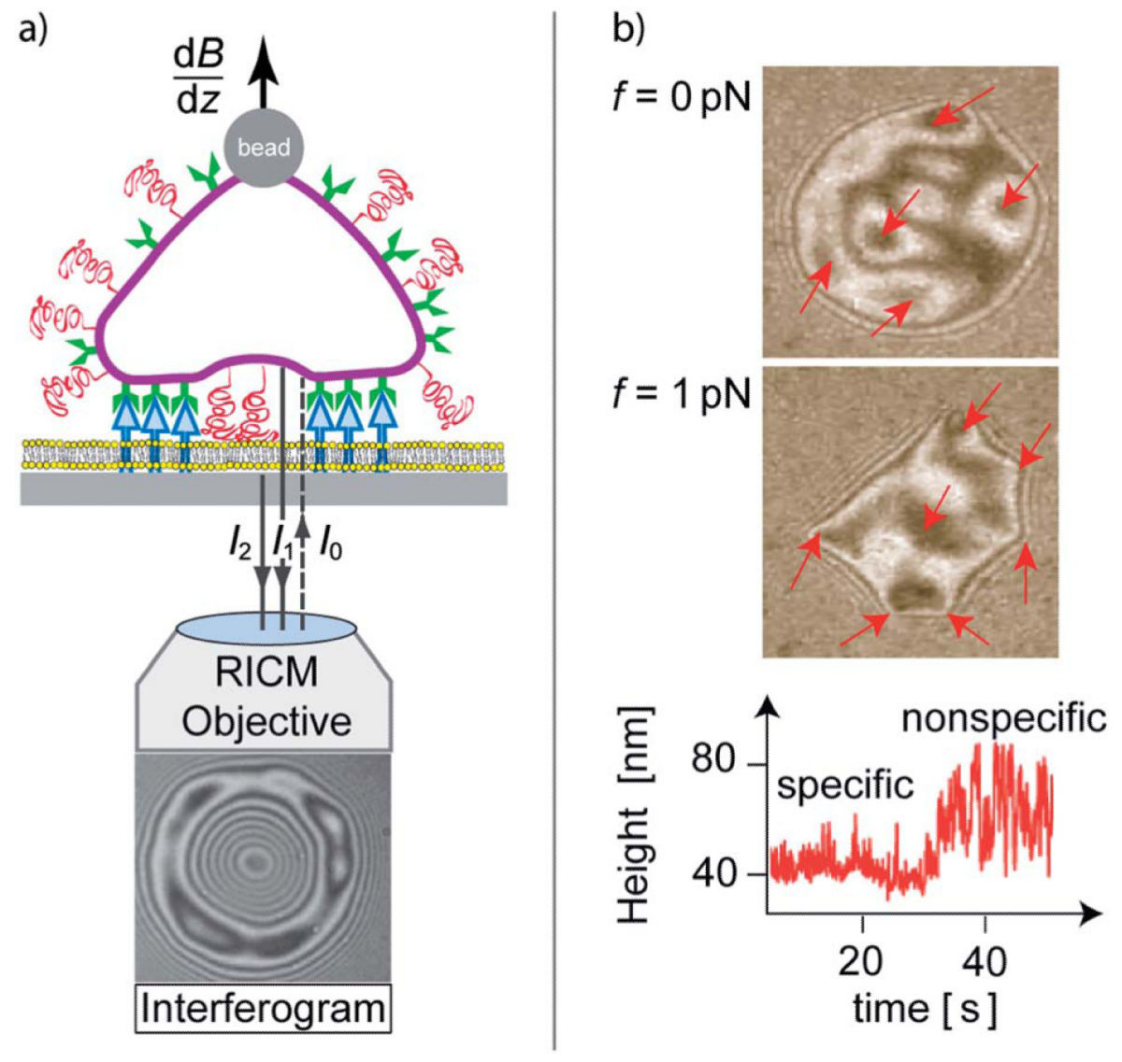
Left: schematic view of image formation by reflection interference contrast microscopy (RICM) by interference of light reflected from the cell (*I*_1_) and the substrate surface (*I*_2_). Lift forces are generated by super-paramagnetic magnetic tweezers subjected to inhomogeneous magnetic fields (d*B*/d*z*). Right: interferogram of a test cell adhering to integrin receptors immobilized on the substrate, prior (top) and while applying a lift force of 1 pN (middle). Some initially visible adhesion domains are indicated by arrows. They are revealed by the formation of dark patches in the absence or of sharp edges in the presence of lift forces. The bottom-right panel shows the time evolution of membrane fluctuations during unbinding of an adhesion domain.

Using supported membranes as test cells or tissue is essential for the application of optical techniques relying on inverted microscopy. The advantage of these techniques and the particular geometry of the mimetic system is that the adhesion zone between a given cell or a vesicle and the functionalised substrate can be observed directly with great accuracy (Fig. 2a). One of the more prominent choices of observation techniques is Refection Interference Contrast Microscopy (RICM),^17^ and its variants Dual and Multiple Wavelength RICM (DW-RICM),^19,20^ which allows for measurements of the absolute height, as well as the Dynamic RICM that maps interface height fluctuations (Dy-RICM).^21,22^ Recently, it has been found that it is possible to combine the benefits of fluoresce and interference approaches within scanning angle interference microscopy.^23^ This family of methods allows us to reconstruct the contour of adhering soft shells close to the surface with 5 nm height resolution, 50 nm lateral localisation precision and with the time resolution of below 10 ms.

The position of randomly formed adhesion domains, hidden within the adhesion zone, can be visualized by several methods. One is the application of lift forces (top panels in Fig. 2b). Alternatively, it is possible to map the membrane fluctuations (bottom panel in Fig. 2b) which then provide dynamic information about domain developments.^24^

## Bending elasticity model of soft shell adhesion

The rigorous elasticity theory of elastic shells is very complex and generally described by the non-linear Föppl von Karman theory (see §11 in ref. 25). Fortunately, the adhesion-induced elastic deformation of vesicles can be described by the simple Helfrich theory of soft shells.^26–28^ This model can also be applied to cells with homogeneous soft shells, such as Dictyostelia,^4^ and blood cells which form small and short lived adhesion domains.^29,30^ The situation is much more difficult for cell types such as fibroblasts that form large stress fibres during adhesion on solid supports.^31^

The elastic free energy can be expressed as:^2^

(1) $$\Delta G_{\mathrm{adh}}\;=\;wA_{c}+\Delta G_{\mathrm{grav}}+\int_{S}\left[\frac{k}{2}{\left(\nabla^{2}h(x)\right)}^{2}+\frac{\sigma }{2}{\left(\nabla h(x)\right)}^{2}+V(h(x))\right]dx$$

Here, the membrane adopting a height profile *h*(**x**) above a plane represented by **x** is parametrised in the Monge gauge. The first term accounts for the free energy of adhesion associated with the formation of specific ligand–receptor bonds. Consequently, *w* is the binding energy per unit area for a specific linker, often referred to as the adhesion strength. *A_c_* is the area of the domain within which the bonds reside. Several approaches were used theoretically to model *w*. In the early studies, the adhesion strength was a phenomenological constant.^32^ Later, by assuming that *w* = ∂Δ*G*_adh_/∂*A*_c_, several microscopic models were derived connecting w to the density of binders and their binding affinity,^33^ as well as to the membrane elasticity and steric interactions.^6^

The second Δ*G*_grav_ term accounts for the gravitational energy and is particularly relevant for the case of vesicles.^34,35^ The last few terms account for the segments of area S of the membrane outside of the domains. More specifically, the third, Helfrich’s term in eqn (1) stands for the energy associated with the bending deformation of the membrane, while the fourth accounts for the membrane tension. The last term takes into account the generic interfacial potential V(*h*), that typically emerges from the short-range steric repulsion and the long-range attraction, with a minimum at intermediate distances of 5–100 nm.^36^ Very often, this potential is approximated by a harmonic form (ref. 2 and 36–38)

(2) V(h)≈∣V0+12∂2V∂h2∣h0(h−h0)2=V0+12γ(h−h0)2

Here, *γ* is the curvature of the generic potential in the minimum positioned at *h* = *h*_0_, and is measured in units of J m^−4^.^10^ This level of description is sufficient for nearly flat membranes, and only recently, corrections in the form of anharmonic terms needed to be implemented to account for the deformation of the membrane that is, due to pinning, taken significantly far away from the minimum of the nonspecific potential.^39^

The four, *a priori* unknown, parameters *w*, *κ*, *σ*, and *γ* can be determined experimentally. The bending modulus *κ* can be determined by micromechanical experiments as performed for vesicles^40^ and cells.^4^ The curvature of the harmonic potential *γ* can be determined by contour analysis of not overly tensed vesicles by noticing that the general free energy functional implies two important length scales: the capillary length *λ* and the persistence length ξ_p_ given by

(3a) $$\lambda =\sqrt{\kappa ∕\sigma }$$

(3b) $$\xi_{p}=\sqrt[{4}]{\kappa ∕\gamma }$$

**Fig. 3:**
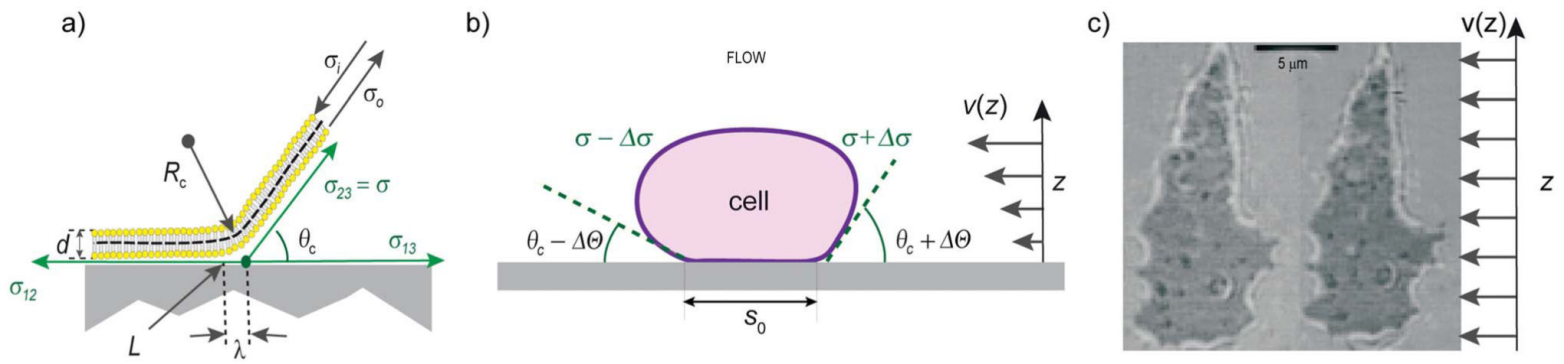
Left: mechanical equilibrium of the interfacial tensions in the radial direction of the adhering shell. The contact line *L*_c_ marks the transition between the adhering and non-adhering zones. The contact tension *σ*_12_ and *σ*_13_ are measures of the interfacial energies between the supported membrane and the vesicle surface and the aqueous phase, respectively. *σ*_23_ = *σ* is the surface tension of the vesicle. Middle: independent measurement of *σ*, *κ* and the free adhesion energies of adhering Dictyostelium cell by contour analysis in the presence and absence of a hydrodynamic flow field. Right: RICM image in the absence (left) and presence (right) of a hydrodynamic flow field. Image adopted from ref. 4. The adhesion strength could be extracted following the discussion in the Appendix of ref. 43.

**Fig. 4:**
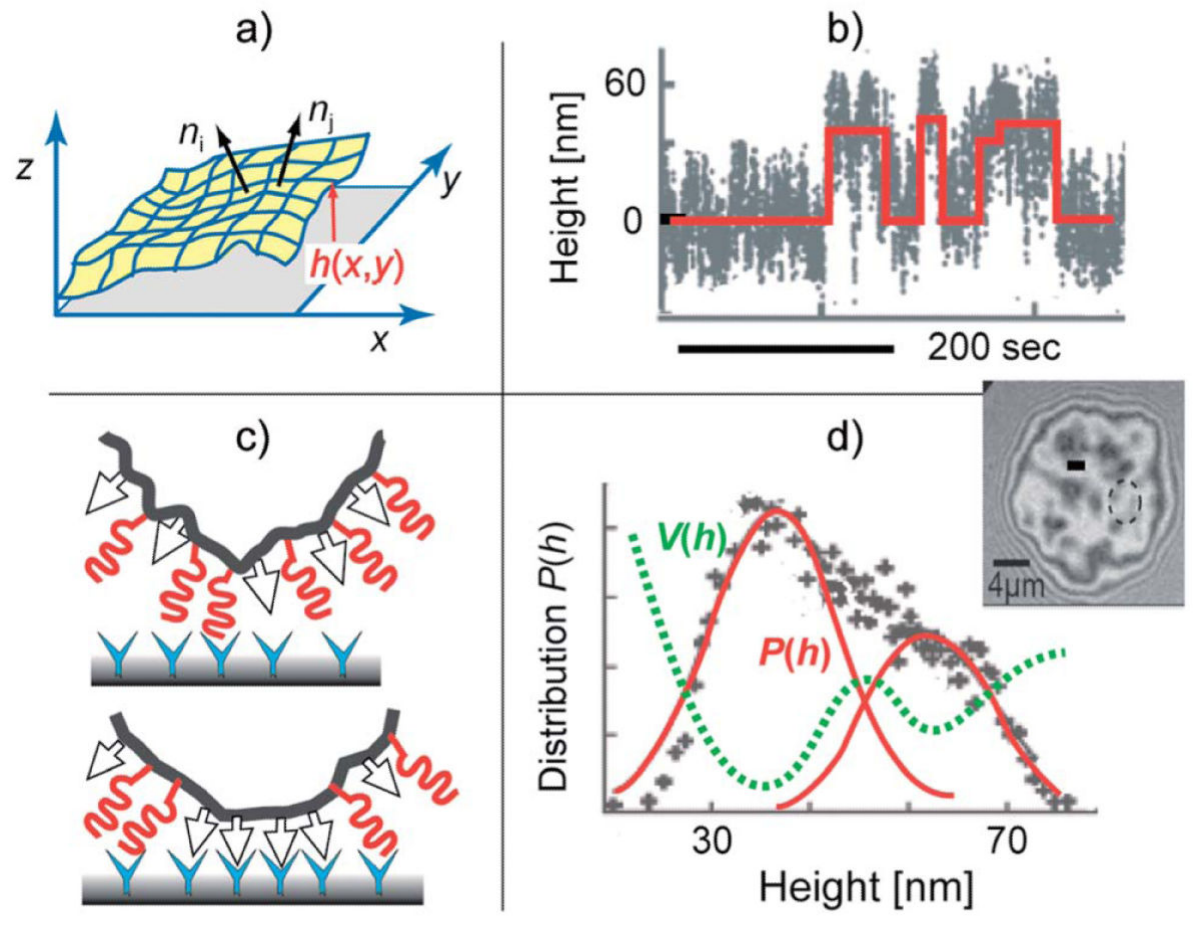
Nucleation of CAM–CAM pairs driven by membrane bending excitations. Top-left panel: characterization of the roughness by the local orientation of the membrane normal that is correlated with the coherence length ζ_p_. Bottom-left panel: formation of CAM–CAM clusters by transient displacement of repellers from the site of the CAM–CAM contact. Top-right panel: time sequence of the distance between the membrane from the solid surface *h*(*t*) at the particular position (measured by RICM at the site marked by a circle in the interferogram). The red line indicates the random transition of the membrane between the bound state 〈*h* 〉 ~30 nm and the unbound state at 〈*h*〉 ~60 nm. Bottom-right: bimodal height distribution *P*f(*h*) defines the double well interfacial interaction potential according to *V*(*h*) ∝ *k*_B_*T* ln *P*(*h*). Image adapted from ref. 41.

The capillary length λ is a measure of the radial distance over which the contour of the shell near the surface is determined by membrane tension (Fig. 3a). The bending deformation evoked by a local point force extends laterally about 2ξ_p_. Both lengths can be measured by analysing the contour of the adhering shell yielding γ.^17^ For example, in weakly adhering, deflated vesicles the capillary length is of the order λ ≈ 1 μm, the persistence length ξ_p_ ≈ 100 nm, and γ ≈ 10^6^ J m^−4^.^36^ Alternatively, weak interaction potentials (strength of the order of 1 *k*_B_*T*) can be reconstructed from bending fluctuations inducing interfacial distance distributions (Fig. 4d).^41^

The contour close to the surface is defined by two geometric parameters: the contact angle *θ*_c_ and contact curvature *R*_c_. For fluid membranes the tensional equilibrium is determined by the well-known Young’s law (eqn (4a)), relating the contact angle *θ*_c_ to the work of adhesion *W*

(4a) $$W\;=\;\sigma (1-\cos\;\theta_{c})$$

The Young’s equation (4a) tells us that the W is a measure of the free energy gained by partial wetting of the surface by the membrane and is analogous to the spreading pressure. Depending on where it is measured, it can be related either to the strength of the nonspecific potential γ, or to the adhesion strength *w* associated with the formation of bonds. Furthermore, the balance of bending moments relates W to the contact curvature,^17^ which provides a condition

(4b) $$W\;=\;\frac{1}{2}\kappa {R_{c}}^{-2}$$

initially derived from the second variation of the free energy with respect to the membrane shape.^32^

By determining the geometric parameters *R*_c_, *θ*_c_ or λ through contour analysis, the free adhesion energy *W* and the surface tension can be measured, provided *κ* is known. Apart from being used in static measurements,^17,41^ this scheme has been applied to dynamic measurements of tension during an adhesion process.^42^ If *κ* is not known *θ*_c_, *σ* and *κ* can be determined by measuring the change of contact angle under hydrodynamic shear flow (Fig. 3b and c).^4^

Alternative approaches including measurements of the adhesion strength of sharp edges formed after application of lift forces (interferogram in Fig. 2b),^43^ or systematic determination of both tension and potential strength from various correlation functions^39^ require a more detailed approach whereby the finite time and spatial resolution of the data acquisition process must be taken into account.^44^

## Modulation of adhesion strength by membrane bending excitations

Lipid bilayers and many cell envelopes (such as red blood cells, macrophages or endothelial cells) exhibit pronounced bending excitations and behave as dynamically rough surfaces (exhibiting roughness amplitudes of several tens of nanometers). According to eqn (3b), local deflections, driven by a point like thermal fluctuations, decay laterally with the persistence length ξ_p_. Thus, fluid membranes can be considered to be composed of square segments of dimensions ξ_p_ × ξ_p_ which perform Brownian motions in the normal direction.^26^ Close to surfaces the collisions of the cushions with the wall exert an entropic pressure, very similar to the 3D pressure exerted by molecules of an ideal gas hitting the wall of a piston. Owing to this analogy, the disjoining pressure between the wall and the membrane placed at an average distance 〈*h*〉 is of the order

(5) $$p_{\mathrm{disj}}∼\frac{k_{B}T}{2\langle h\rangle \xi_{p}^{2}}$$

The dynamic roughness of the membrane emerges as a mean square deviation 〈Δ*h*^2^〉 from the mean profile 〈*h*(**x**)〉, where the instantaneous profile is given by *h*(**x**, *t*) = 〈*h*(**x**)〉 + Δ*h*(**x**, *t*). It can be characterized quantitatively by integration over all bending modes ***q*** ≡ (*q*_1_, *q*_2_)

(6) $$\langle \Delta h^{2}\rangle \;=\;\frac{k_{B}T}{{\left(2\pi \right)}^{2}}\int dq\frac{1}{\kappa q^{4}+\sigma q^{2}+\gamma }$$

with *q* ≡ |***q***|.

For membranes residing in a harmonic potential, and in the limit of zero tension, the dynamic roughness and the disjoining pressure are given by the celebrated Helfrich equations

(7a) $$\langle \Delta h^{2}\rangle {|}_{\sigma =0}=\frac{\kappa_{B}T}{8\kappa }{\xi_{p}}^{2}$$

(7b) $$p_{\mathrm{disj}}\approx c\frac{{\left(k_{B}T\right)}^{2}}{\kappa {\langle h\rangle }^{3}}$$

where the pre-factor *c* = 0.23 is determined from Monte Carlo simulations.^45^

The dynamic disjoining pressure attenuates the local non-specific interaction potential *V*, changing both the curvature and the position of its minimum. Following eqn (2) and (4a), the effective energy of the adhered state is shifted to higher energies^2,6^

(8) V(h0)≈∣V0+12∂2V∂h2∣h0〈Δh2〉=V0+kBT8κγξp2

The curvature of the harmonic potential for weak adhesion (*V*_0_ ~ 10^−6^ J m^−2^) is of the order of *γ* ~ 10^14^ J m^−4^.^10^ With ξ_p_ ~ 25 nm and *κ* ≈ 10^−19^ J we expect a shift of the potential minimum *V*(*h*_0_) to 10^−5^ J m^−2^.

If adhering shells are subjected to strong membrane tensions *σ*, the long wavelength excitations of the membranes are suppressed and the roughness is drastically reduced to

(9) $$\langle \Delta h^{2}\rangle \;=\;\frac{k_{B}T}{2\pi \sigma }\ln\left(\frac{\sigma }{\sqrt{\kappa \gamma }}\right)$$

The suppression of the dynamic roughness by membrane tension^36^ triggers the transition of soft shells from weakly to strongly adhering states, in a process called tension-induced switching of adhesion.^17,46^

The non-specific interactions control cell adhesion in various ways. First of all, they impede the non-specific attachment of cells to other cells or tissues, which has been shown explicitly for several cell types, including blood cells (erythrocytes and macrophages).^29,30^ In the case of erythrocytes, prevention of non-specific adhesion has been achieved by actively enhancing fluctuations.^47^

The non-specific interactions, furthermore, couple to the specific intermolecular recognition promoted by ligand–receptor interactions. In the context of equilibrium, this coupling affects the affinity of binding.^48^ If ligand–receptor bonds form domains, the effect of specific binding can be represented by an effective adhesion potential. The change of this potential due to the steric repulsion can be treated within the same mean-field approach outlined above, and a shift of the potential depth, which depends on the affinity of binding, can be predicted. For example, for intrinsically strong bonds such as biotin–streptavidin packed in densely packed domains (*V*_0_ ~ 10^−5^ J m^−2^), the bending excitations play a negligible role. However, membrane fluctuations may strongly affect the integrin–RGD bonds for which *V*_0_ ~ 10^−7^ J m^−2^,^9^ or bonds in sparsely packed domains. Beyond this scaling approach, an effective binding affinity for a given ligand–receptor pair can be calculated more rigorously as a function of the properties of the membrane and the nonspecific potential.^6^

In the context of dynamics, bending excitations play a key role in the nucleation of adhesion domains,^49^ and drive the contact formation between CAM–CAM pairs of cells. This was recently rationalised through binding and unbinding rates calculated as averages over typically fast membrane fluctuations,^50^ yielding an accurate model for the nucleation of adhesion domains, as verified by high-level Langevin simulations.^51^ The importance of membrane fluctuations for the nucleation of adhesion domains has also been shown in the cellular context.^52^ Thereby however, expulsion of the glycocalyx repeller† molecules from the nucleating domains may play an additional role (Fig. 4).

## Soft shell adhesion as a heterogeneous wetting process

**Fig. 5:**
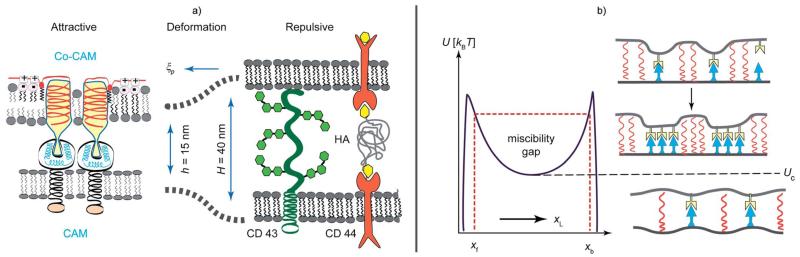
Left panel: schematic view of three major forces controlling the primary phase of cell adhesion – (i) the short range attractive lock-and-key forces between CAM molecules act within the typical range of *h* ~ 15 nm. (ii) The repulsive forces mediated by membrane proteins with large extracellular domains (for example CD 45, CD 43 and ICAM) or hyaluronic acid (HA) molecules anchored to membrane receptors of the CD 44 family. (iii) Elastic stress caused by the length mismatch (*H* ≠ *h*). The range of the deformation is determined by the persistence length ξ_p_ (eqn (3)). Right panel: phase diagram for adhesion. The ordinate shows the normalized bending energy and the abscissa the volume fraction of ligands. *U*_c_ marks the lower critical point of the miscibility gap. Above *U*_c_, long range attraction of the isolated CAM–CAM pairs leads to the formation of microdomains. Bellow *U*_c_ a homogeneous state appears, which is realized for very low CAM concentrations and small height mismatches (*H* − *h* ≈ 0).

Interferograms in Fig. 2 depict a typical situation of a vesicle doped with small concentrations of attractive and repellant linkers adhering on the supported membranes containing attractive CAMs. The adhesion zone decays into domains of strong specific adhesion and regions where the membrane rests in the minimum of the weak non-specific potential. The linker condensation is driven by the cooperative effects arising from membrane elasticity and fluctuations.^53^ Additional contributions are elastic stresses arising in the membrane if the interfacial equilibrium distances of the bonds (*h*) and the length of the repellers (*H*) are not matched (Fig. 5).^54,55^ Since many cells (red blood cells, macrophages, endothelial cells) exhibit pronounced, sometimes actively driven, bending excitations this effect is of primary biological relevance.^29,52^ Lateral bond condensation can also be diffusion driven by the direct short range attraction between CAMs.^53,55^

## Basic rules of physics of cell–cell adhesion learned from model membrane studies

### Adhesion domain stabilisation by actin cortex

Cell adhesion starts with the rapid formation of micro-domains of tight contact formed by diffusive segregation of bound pairs of cell adhesion molecules (diffusivity: *D* ≤ 0.1 μm^2^ s^−1^). Resting cells expose typically ~10^4^ receptors. Clusters of linkers could form in fractions of seconds. The nucleation of CAM clusters is further accelerated by pushing forces generated by membrane bending excitations.^20,22^ In a secondary step, the intracellular domains of the CAMs bind to the actin cortex, resulting in the stabilization of the adhesion domains^56^ by two mechanisms:

The first mechanism consists of the increase of integrin binding affinity for ligands in the extracellular matrix, mediated by coupling of talin with its FERM domain to the β-chains of integrins. The adhesion strength, measured in terms of the spreading pressure *W* (eqn (4)), increases by about a factor of 5 due to the increased bending stiftness of the cell envelope.^4^ In the resting state of cells, talin resides in the plasma membrane in the sleeping conformation. Activation by phosphorylation^57^ exposes the FERM domain and talin is recruited to the membrane by binding to PIP2/PIP3 *via* pleckstrin homology domains and electrostatic–hydrophobic forces. Talin tends to form dimers^57^ and exhibits several actin binding sites at the C-end. It can thus form gel patches and stabilize the adhesion domains.

**Fig. 6:**
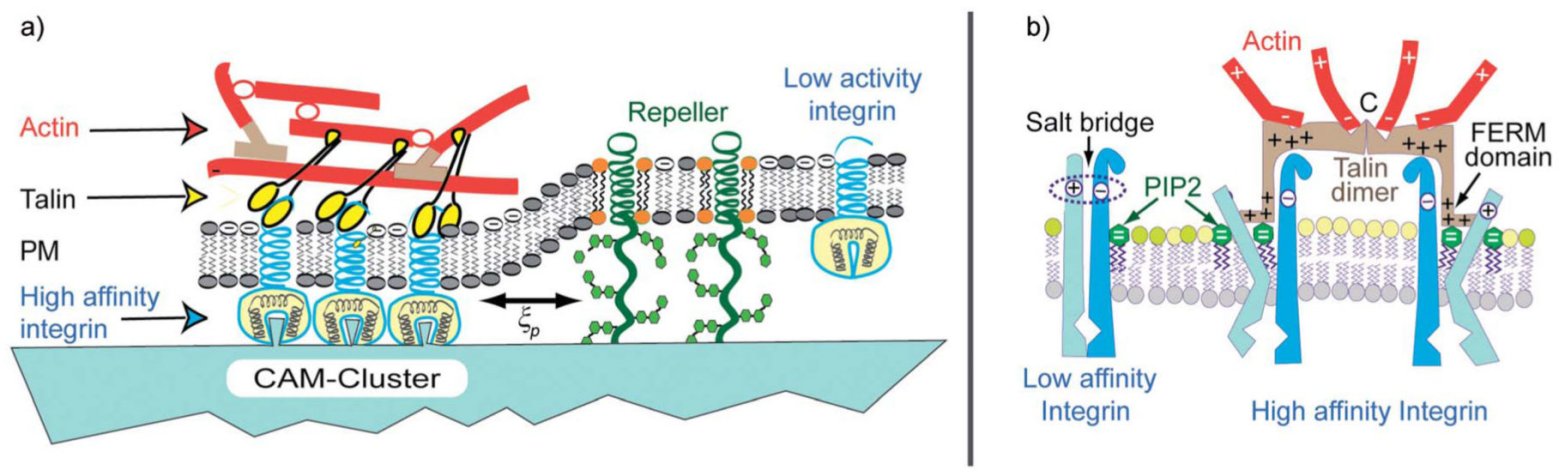
Left panel: coupling of actin gel patches to a CAM cluster of the integrin family by talin. The latter is associated with a dramatic increase of the integrin binding affinity. Right panel: molecular model of integrin activation by binding of the talin FERM domain to its *β* chain, resulting in the opening of the integrin binding pocket. The increase in affinity is mediated by the binding of the FERM domain of talin that uncouples the salt bridge between the integrin intracellular domains. Moreover, the FERM domain is also directly coupled to the membrane by electrostatic forces and phosphoinositides (PIP2/PIP3). Talin which forms dimers links two integrins and has several binding sites for F-actin. Thus actin gel patches can form without contribution of other actin cross-linkers. As a consequence, the formation of adhesion domains could be even mediated by local actin gelation.

A second effect of talin is the increase of the affinity of talin by opening their binding pocket by a mechanism shown in Fig. 6. In summary, talin is a major driving force for the clustering of receptors and the formation of adhesion domains within cells.

### Control of cell adhesion by receptor inhibitors

A biologically important control mechanism of the cell tissue adhesion consists of blocking the binding sites for CAMs on the tissue surface. The physical basis and consequences of this mechanism has been studied using biomimetic systems.^24^

**Fig. 7:**
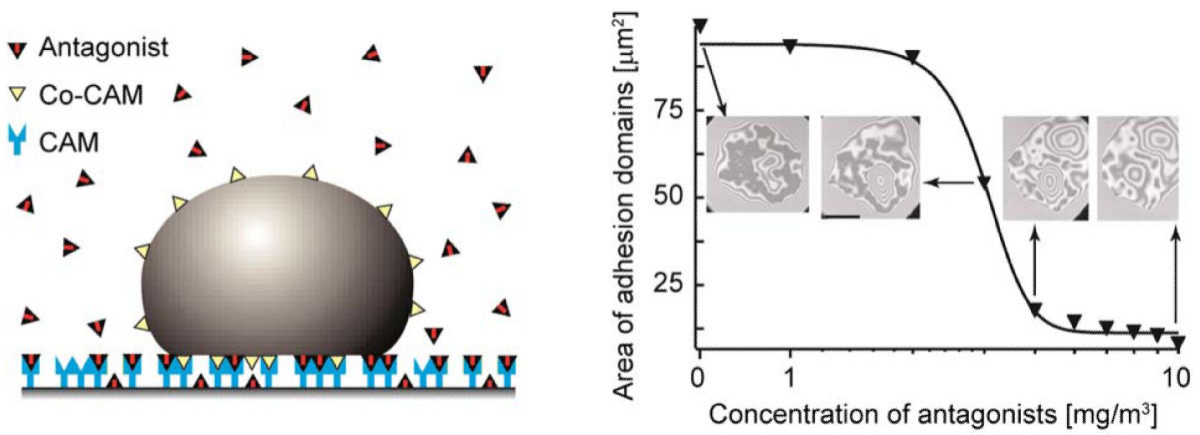
Schematics of the model system used to study the control of the size of adhesion domains formed of CAM/Co–CAM bonds by competitive antagonists that inhibit CAMs.^24^ The graph represents the total area associated with tight adhesion domains within the adhesion disc, plotted as a function of antibody concentration in bulk solution. Dark areas in interferograms should be associated with adhesion domains.

The vesicle is first allowed to reach a steady state adhesion mediated by ligand–receptor recognition. After this state is achieved, soluble antibodies for CAMs are inserted into the system. Two mechanisms of response to the presence of antagonists have been identified. In the fast response, the antibodies exert a lateral pressure on domains consisting of ligand–receptor complexes, resulting in an immediate shrinking of domains. After this pressure is equilibrated, the antagonist penetrates the domains and the second, slower response takes place. Thereby antagonists competitively bind and block CAM molecules within the domain upon stochastic unbinding of the ligands (Fig. 7). Because the antagonists have a stronger affinity to the CAM than the ligands, the ligands in the membrane cannot rebind, and the domain may ultimately become unstable.

The most intriguing result of this experiment is the threshold behavior of the antagonist-mediated abolishment of the adhesion, which can be explained quantitatively by a thermomechanical adhesion model.^24^ The total number of ligand–receptor bonds decreases first weakly with increasing concentration of the antagonist but drops abruptly by about a factor of 100 within a narrow concentration regime at the threshold antagonist concentration (Fig. 7). Upon this drop, a saturation regime is reached where the concentration of antagonists has to be increased further for a few orders of magnitude to combat the thermodynamic response of the vesicle that now contains a large number of free ligands. These have high probability to find a free CAM molecule and establish a bond, thus preventing a complete unbinding of the target cell-mimic.

The behavior discovered in cell-mimetic studies can provide an explanation for the enhanced penetration of metastatic cancer cells through tissue. Cancer cells are known to over-express metal proteases that cleave extracellular segments of the repellant glycoproteins. These segments bind specifically to extracellular matrix proteins acting as antagonists of specific CAMs (such as integrins). They can thus impede the adhesion of cells in tissues, which facilitates their progression within the body. The finding of threshold behavior in our cell-mimetic experiments (see Fig. 7) suggests that a possible difference between normal cells and metastatic cells is that the former are below, and the latter above the unbinding threshold.

The critical concentration depends sensitively on the ratio of the affinities of the ligands and antagonists for CAM molecules. It is worth noting that soluble ligands themselves can be antagonists for the membrane confined ligands, simply because of their higher affinity for CAMs. This difference in affinities emerges from the conformational space of the ligand by confining it to the membrane^48^ and because of stochastic excitations that the fluctuating membrane exerts on the bonds.^6^

### Comparing cell–cell with cell–matrix adhesion: adhesion strength, internal domain organisation, and response to force

**Fig. 8:**
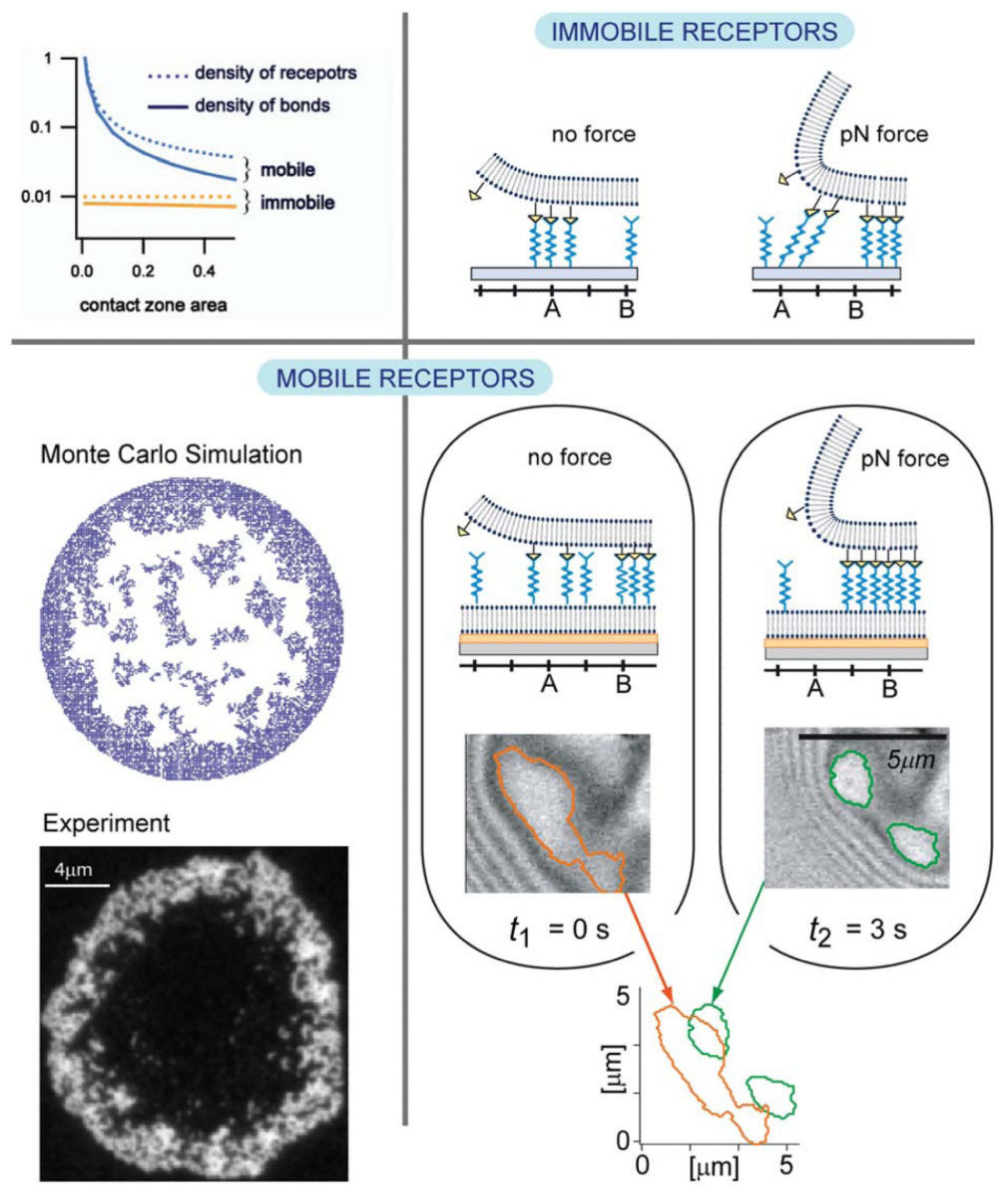
Top-left panel: density of receptors and bonds as a function of the size of the adhesion zone. When mobile, the density of receptors increases despite the fact that the density in the bulk of the bilayer is kept constant. Bottom-left: ring-shaped self-assembled adhesion domains form due to the interplay of diffusion and affinity of binding. Top-right panel: schematic representation of the domain response to the force when the receptors are immobile. The bonds are stretched, break and reform on new receptors in the interior of the adhesion zone. Bottom-right panel: schematic representation of the domain response to the force when the receptors are mobile. The domains densify in response to the force by displacing intact bonds for up to several microns.

Comparative studies of giant vesicles adhering on target membranes containing mobile and immobile CAMs (integrins) revealed fundamental differences between cell–cell adhesion, where both linkers are mobile, and cell–extracellular matrix adhesion where the co-receptors are immobile. First, the total area of tight adhesion and the spreading pressure W of each domain are much higher for mobile CAMs.^3^ This emerges from the fine balance between mixing entropy and binding enthalpy that determines the steady state of adhesion. In the case when both binding partners are mobile, the number of bonds is significantly higher than in the case when one of the partners is immobile, an effect that is further enhanced for small contact areas of test cells with substrates (top left in Fig. 8).

Not only the number of bonds but also the organisation of bonds changes dramatically with mobility of binding partners. Unlike in the case when the distribution of bonds within the domain is imposed by the spatial organisation of the immobile CAMs, establishment of bonds between two mobile CAMs allows for the optimisation of the inter-bond distances. The organisation of bonds within the domain depends intimately on elastic stresses promoted by the fluctuating membrane. The latter are responsible for cooperative effects between bonds,^58^ but also decrease the affinity of individual bonds, hence, the overall balance of enthalpy and entropy.

Our current understanding is that only relatively high densities of immobile receptors support the formation of domains. At very high densities, large circular domains are obtained often filling the entire vesicle–substrate contact zone. Decreasing the density yields irregular dendrite like structures even if the kinetics is reaction limited.^38,59^ On the other hand, in the system with mobile receptors, stable domains form even at biologically relevant low densities. In this case, both densely and dispersedly packed domains are found to coexist^60^ in a regime of intermediate and low binding affinity when a free energy barrier separates the two packing states.^6^ At large binding affinities, the enthalpy dominates, and the system attempts to maximise the number of bonds and only dense packing is expected. However, high affinity also promotes fast recognition and a long life-time of the CAM–CAM complexes, which are typically immobile. Consequently, existing bonds become obstacles for CAMs that are recruited from the outside of the contact zone, and jammed structures corralling the contact zone may emerge (bottom left in Fig. 8).^61^

Lastly and maybe most importantly, the two systems respond very differently to external forces (right panels in Fig. 8). If subject to a constant force, the density of bonds in the reduced vesicle–substrate contact zone increases to re-establish thermomechanical equilibrium (Fig. 7, left).^62^ However, the mechanisms leading to the densification are very different depending on the mobility of CAMs. If an external lift force is applied to vesicles adhering on a target with fixed CAMs, the bonds located at the edge of the contact zone are stretched and eventually break, reducing the contact area (top right in Fig. 8). New bonds form in the remainder of the contact zone preventing further detachment. However, the densification is restricted by the distribution of the immobile CAMs and the vesicle may unbind under relatively low force. Because the change in the spreading pressure between the equilibrium under force and the equilibrium without the force, releasing the vesicle is nearly a reversible process.

In striking contrast, within seconds after switching-on the force, the bonds close to the contact line move laterally without unbinding up to micron distances, and sparse domains restructure into densely packed structures that may be enforced by new CAMs which join due to cooperative effects. Consequently, the adhesion strength of domains increases under stress (bottom right in Fig. 8), and the unbinding force necessary to lift off the vesicle grows by several orders of magnitude.^63^ Because of this increase of adhesion strength, the transient spreading pressure induced by the force-driven condensation of CAM–CAM pairs results in the monotonous growth of the contact zone if external forces are applied repeatedly.

The force induced strengthening of mobile linkers is very fast (<1 s). This can play an important role in the sub-second adaption of cells to changing elastic stresses in tissue before the cell can respond by reorganization of the actin cortex. Under physiological conditions, this adaption is mediated by the reorganization of the actin cortex and microtubule–actin crosstalk and can take minutes as discussed in the following sections.

## Biological paradigms of cell adhesion

### Immunological synapses

#### Stimulation of T-cell proliferation by biochemical reaction centers

**Fig. 9:**
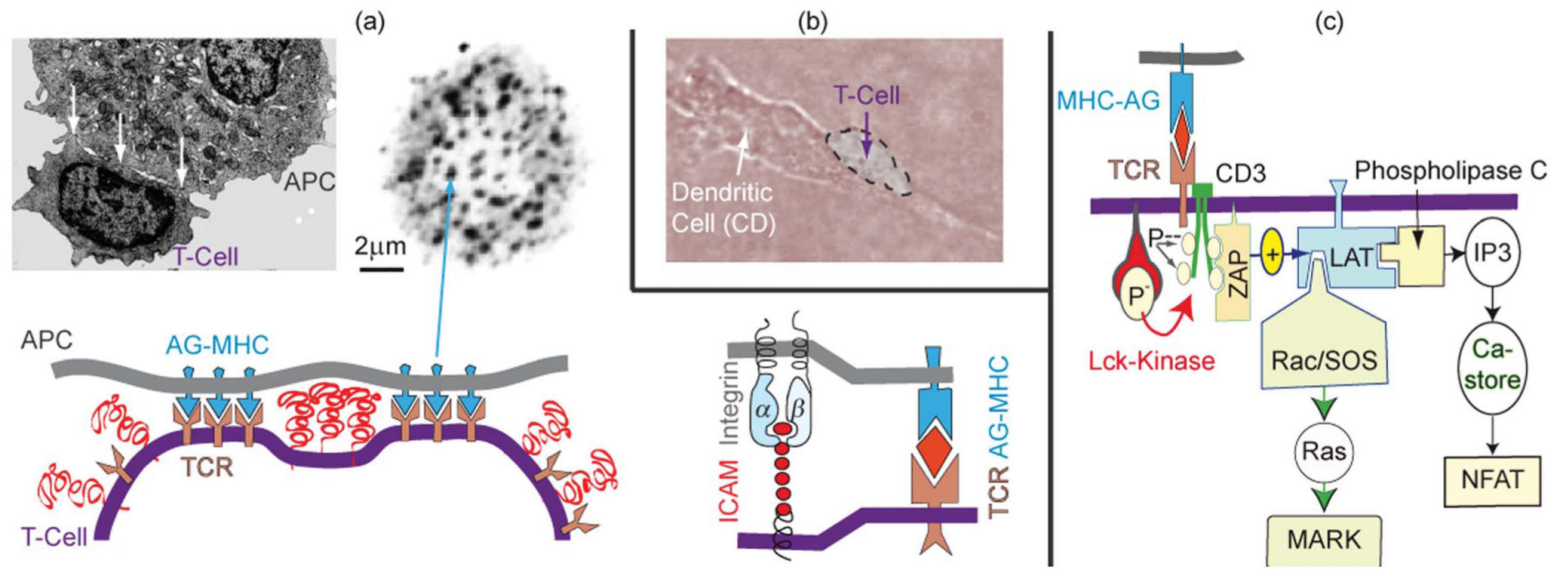
(a) Top left: electron micrograph showing T-cell adhering on antigen presenting cells (APCs) by generating domains of tight adhesion (see arrows) separated by unbound zones. Top right: direct visualization of adhesion domains by fluorescence labeling of the T-cell receptor (after ref. 3). Bottom left: schematic view of adhesion-induced domain formation. Bottom right: the adhesion domains are possibly formed by co-aggregation of TCR–AG–MHC and integrin–ICAM-1 linker pairs as discussed in ref. 7. (b) Phase contrast micrograph of a polarized T-cell adhering and migrating on the antigen presenting dendritic cell. (c) Simplified scheme of genetic expression of the cytokine interleukin II (IL-2) by binding of the MHC–AG complex to the T-cell receptor (TCR) which is tightly associated with the co-receptor CD3. The binding triggers the phosphorylation of the 4 tyrosine groups at CD3 which results in the attraction and activation of the effector ZAP. The excited ZAP triggers the activation of the strongly membrane-bound scaffolding protein LAT. This activated scaffolding protein recruits activator and adaptor proteins including the phopsholipase Cg and SLP76. The former induces the genetic expression through the transcription factor NFAT. SLP76 mediates the activation of the genetic expression via the MAPK mediated pathway. Both pathways must be activated to express IL-II and to elicit a true immune reaction. The image on the left panel of (a) and the phase contrast micrograph was reproduced from ref. 7.

A vital biological process is the stimulation of freshly born (=naïve) lymphocytes (T-cells) by encounters with antigen presenting cells (APCs, see Fig. 9a), such as dendritic cells (DC). The naïve T-cells move from the bone marrow to the lymph node where they encounter specific antigen exposing cells, called dendritic cells.^1^ Sequential or continuous adhesion of the T-cells on the APC stimulates the production of cytokines, such as interleukin II (IL-2), which eventually leads to the division of the generating cell and other T-cells forming clones.^65^ The stimulation can also be mimicked under *in vitro* conditions. First, by embedding the DC in collagen networks where they are encountered transiently by the naïve T-cells (see Fig. 9b) and second, by mimicking DC by supported membranes doped with antigen–MHC complexes.^66^ In both cases the T-cells are stimulated after >12 h.

**Fig. 11:**
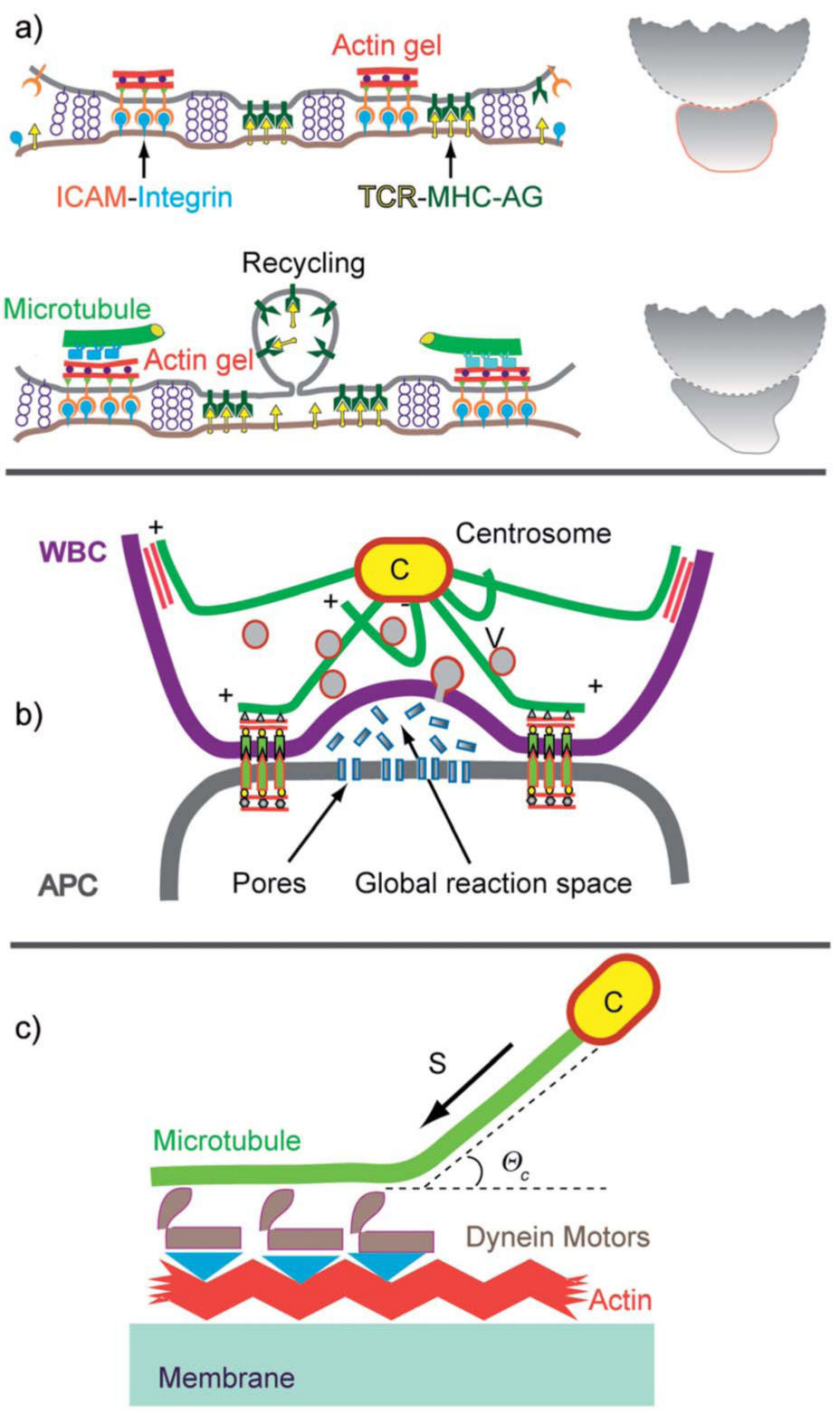
(a) Reorganisation of the T-cell–APC adhesion zone (left) and cell shape (right) suggested by visualisation of talin and Lck distribution.^71^ The top image shows the situation 3 min and the bottom 25 min after contact formation. The right panels show the contours of the cells redrawn from phase contrast micrographs.^67^ (b) Dome-like reaction space formed by cytotoxic T-cells adhering on infected target cells. The global shape is stabilized by microtubules linking the actin cortex to the centrosome (C). A second fraction of MT exhibits dangling plus ends which serve as tracks for the rapid transport of secretory vesicles and endosomes by dynein and kinesin motors.^72^ (c) Model of mechanical stabilisation of the cell shape by tangential coupling of MT to the actin cortex by dynein motors.^73^

The physical basis of the process has been described in detail previously.^7^ We therefore summarize here only results to demonstrate that adhesion domains play a key role as local reaction platforms that promote the expression of cytokines by the coordinated activation of two transcriptional pathways. The electron micrograph of a T-cell adhering to a virus infected cell shows that the adhesion zone is not homogeneous but decays into patches of tight adhesion (see Fig. 9a). The adhesion domains are formed by lateral aggregation of antigen–MHC-II complexes (AG–MHC) bound to T-cell receptors (TCR). Electron microscopy studies based on immune labeling with antibodies to talin^67^ show that adhesion is also driven by formation of integrin–ICAM-1 links. The binding constant of ICAM-1–integrin pairs is of the order *K*_d_ ~ 100 nM (corresponding to a binding energy of *w* ≈ 7 *k*_B_*T*), while that of TCR–MHC–AG complexes is much weaker and lies around 10 μM (*w* ~ 4 *k*_B_*T*).^68^ It is therefore possible that the ICAM–integrin pairs drive the adhesion and may even aggregate together with the TCR–AG–MHC pairs (Fig. 11).

#### Adhesion domains as biochemical reaction centers

The adhesion domains formed by T-cell adhering on antigen presenting cells (APCs) such as dendritic cells^66,67^ are a condition *sine qua none* for the T-cell activation. It is triggered by phosphorylation of the tyrosine kinase receptor CD3 by the membrane anchored kinase Lck which is, however, constantly counteracted by the conjugate phosphatase CD45. Owing to its large extracellular domain, it can only dephosphorylate CD3 if the lymphocyte is free. Its function is inhibited after formation of adhesion domains since it is expelled from these reaction platforms, exhibiting an interfacial distance of ~15 nm interfacial distances.^69^

#### Global reaction space generation by cell polarization and actin–microtubule crosstalk

Some 10 minutes after T-cell–APC encounters the adhesion zone reorganizes. A ring-like tight adhesion zone of integrin–ICAM-1 bonds forms at the contact line,^66,67^ enforced by the influx of new I-CAMs from the top of the T-cell^70^ and the increase of the integrin binding affinity by talin binding (Fig. 6). The contact area increases by ~20%. The T-cell polarizes assuming a pear-like shape. The polarized shape is stabilized by coupling of the microtubule plus ends to the actin cortex (Fig. 11c). The TCR–AG–MHC domains move to the center of the adhesion disc and form large complexes. These “supra-molecular activation clusters” (SMACs) facilitate the recycling of the immune synapses by endocytosis.^64,69^

The secondary process generates a closed reaction space which facilitates the destruction of infected cells by cytotoxic T-cells (middle panel in Fig. 11b). The target cell is destroyed by pores generated by lytic peptides, such as perforin, secreted by the killer cell. The ring-like gasket prevents the escape of the proteins into the extracellular space.

Previously, we showed that domelike spaces can be stabilized by the balance of the membrane tension generated at the inner and outer contact line of the adhesion ring.^51^ Furthermore, corrals constructed by CAM–CAM bonds may spontaneously form in a self-assembly process (bottom left in Fig. 8). However, the cell shape is mainly stabilized by coupling of the microtubule plus ends to the actin cortex.^73,74^ The MT–actin coupling can be mediated by specific binding proteins or by dynein motors coupled to actin as shown in Fig. 11c. In the former case a one-dimensional spreading pressure S pushes the MT towards the rim of the dome. In analogy to the spreading pressure of adhering membranes, the tensile force *Σ* can be expressed as *w* = *Σ*(1 − cos *Θ*_c_). Here, *w* is the binding energy per unit length of MT and *Θ*_c_ is the contact angle. In the second case the pushing force *F*_R_ of the MT towards the contact line is generated by the dynein motor tendency to walk towards the centrosome.

### Endothelial cell layers and the dynamics adhesion of rolling and transmigrating

**Fig. 12:**
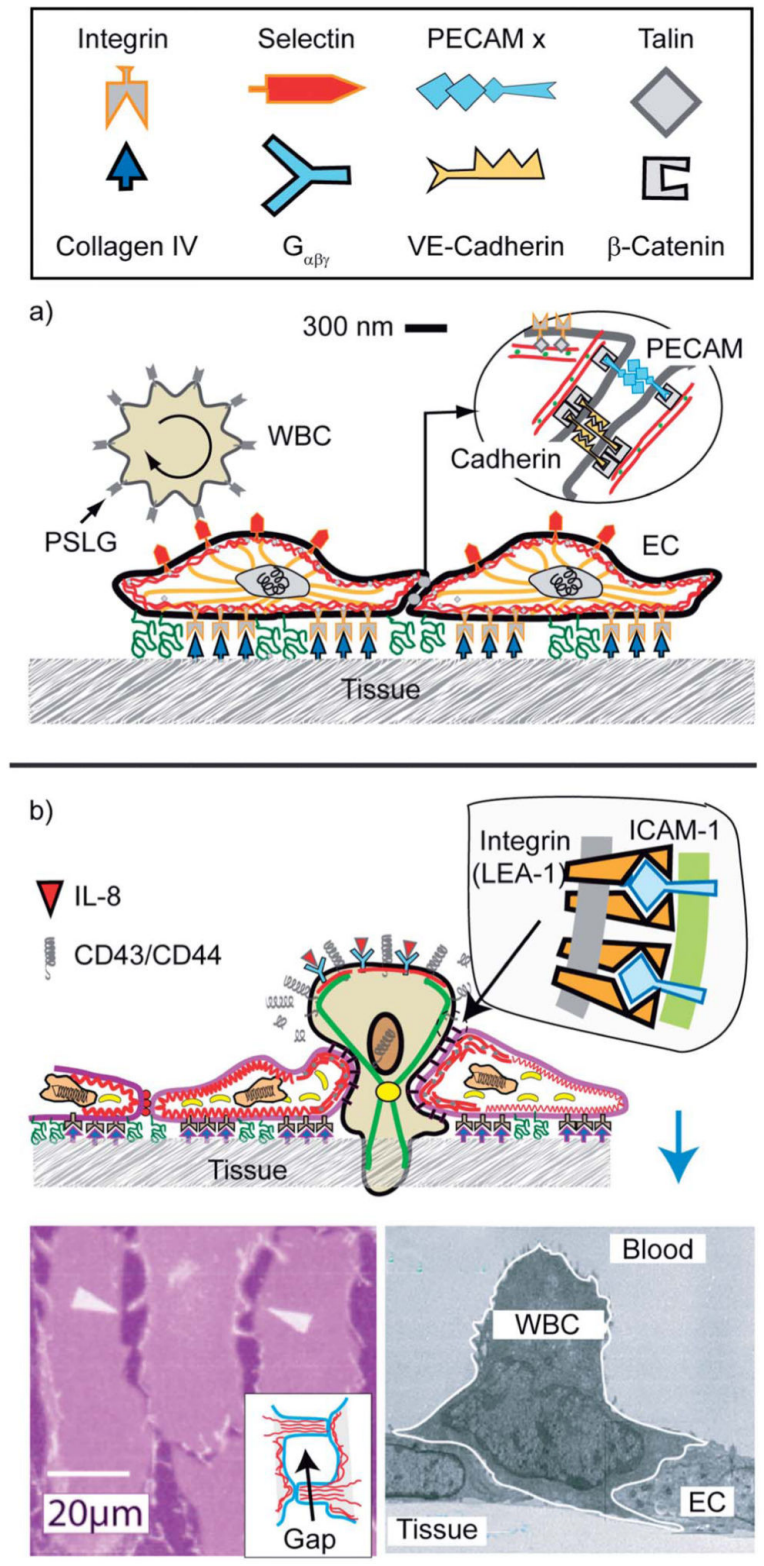
Schematic view of endothelial cell monolayers with adhering WBC in the resting state. The leucocyte (WBC; shown on the left side) exposes ~5000 microvilli (length 0.3–0.5 μm; width 150 nm).^76^ The number of PSLG-1 receptors (5 × 10^5^) is 100 times larger than that of microvilli, suggesting that the tip is coupled to several PSLG-1–selectins bonds. The WBCs expose receptors for cytokines (abbreviated as G_αβχ_) which activate the cell through the heterogenous membrane bound GTPase G_αβγ_. (b) Reproduced from E. Sackmann and R. Merkel, *Lehrbuch der Biophysik*, Wiley Verlag, Weinheim, 2010. Penetration of the excited lymphocyte through the ENC-layer triggered by cytokines (such as interleukin-8). They bind to specific receptors on the blood cells which increases the density of high affinity integrins LFA-1 on the WBCs while the repellant glycoproteins are removed from the front. Bottom left: electron micrograph showing activated cell (granulocyte) penetrating through the endothelial cell monolayer (reproduced from ref. 77) The driving force is provided by the gain in binding energy between integrin (LFA-1) and ICAM. Bottom right: opening of a gap between ENC monolayers by the hormone histamine and thrombin.^78^

The migration of white blood cells through endothelial cell layers discussed in this section is an example of the biological relevance of large scale phase segregation of attractive and repellent CAMs and the cell polarisation by the accompanying reorganisation of the actin cortex. The barrier between blood and tissue is formed by confluent monolayers of endothelial cells (ENC) lining the inner wall of blood vessels. The endothelium is stabilized (i) by cell tissue adhesion through binding of integrins to proteins of the basal membrane (such as collagen IV), and (ii) by cell–cell adhesion mediated by the self-recognizing (homophilic) CAM cadherin and PECAM (Fig. 12). The co-cluster of cadherin and PECAM,^75^ together with the membrane bound receptor of the growth factor VEGF, forms a stress sensor that plays a key role in the adaption of the cell tissue adhesion strength to the varying hydrodynamic shear forces in blood vessels.^79^

A key adhesion controlled process considered here is the enforced permeation of leucocytes through the walls of the vessels at sites of inflammation. Most of the time, the cells patrol the body by rolling along the surface of the endothelium.^76^ The cells are locally coupled to the endothelial cells (EC) by specific binding between CAMs of the selectin family on the ENC surface and the glycoprotein PSLG-1 accumulates on the tips of the ~0.5 μm long microvilli. PSLG-1 exhibits a FERM binding domain and can thus couple to the actin filaments penetrating into the villi, *via* moesin or ezrin.^80,81^ In this way the WBC–ENC bond strength can be rapidly adapted to shear stress (*Σ*) fluctuations by changing the area of contact between microvilli and the ENC.^80^ This adaption can be explained in terms of the force induced increase of the linker density as demonstrated by biomimetic model studies (see Fig. 8).

**Fig. 13:**
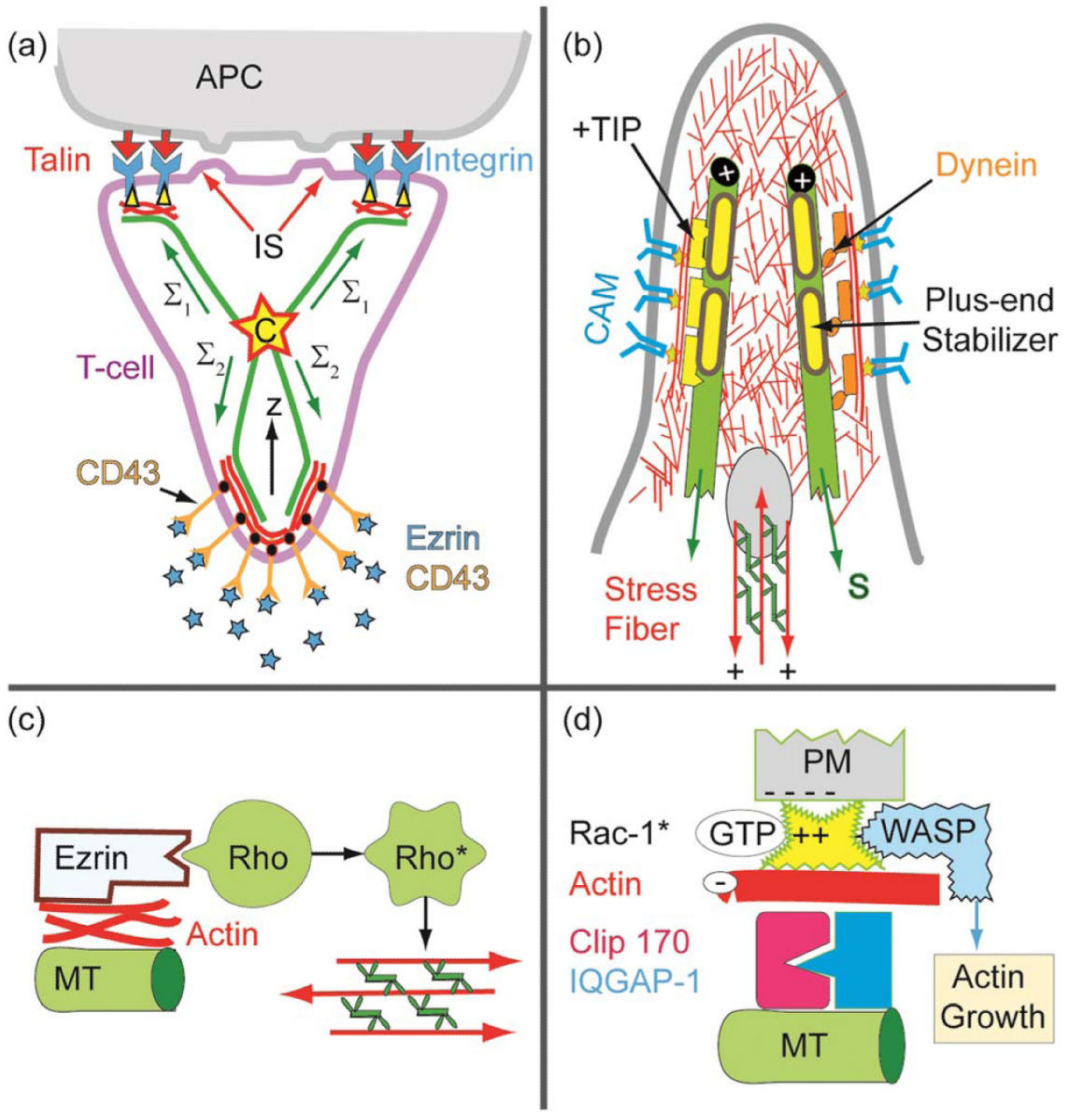
(a) Model of bipolar polarization of T-cells moving in lymph tissues and encountering an antigen exposing dendritic cell (see BOX Fig. 1a). Note that the mechanical cell stability is determined by the balance of the traction forces in the MT which can be generated by passive MT–actin coupling or dynein motors (Fig. 11c). (b) Detailed view of rear of polarized cells showing the MT–actin coupling by passive linkers (left) and dynein motors (right). The yellow bars on the MT plus ends stand for regulators of the actin polymerization such as the IQGAP/Clip 170 complex which activates the actin gelation (see (c)). (c) Mechanism of F-actin–MT coupling via the complex Clip 170/IQGAP1, which can recruit and activate Rac-1 which in turn activates the actin polymerization promotor WASP.^82^ (d) Activation of Rho-A GTPase coupled to the ezrin–MT complex. GTP-Rho-A triggers the activation of the myosin-light chain kinase (MLCK), resulting in the self-assembly of stress fibers (micro-muscles) which are coupled to adhesion domains.

A dramatic change of behavior of the WBC occurs at sites of inflammation by binding of cytokines to G_αβγ_ linked receptors, triggering the increase of the integrin (LFA-1) affinity (Fig. 13a). The cell becomes polarized with the front adhering strongly on the surfaces of adjacent ENC by binding of the high affinity integrin LFA-1 to ICAM-1, while the repellant selectins are cleaved at the front by proteases or move to the trailing end. Interestingly, the density of high affinity LFA-1 linkers is drastically increased by exocytosis of vesicles loaded with LFA and proteases.^1^ The polarized state of the cell is stabilized by cross-talk between actin and microtubules (Fig. 13b).

### Cell polarization by actin–microtubule crosstalk

The biological examples shown above confirmed the important function played by repellant CAMs for the control of cell adhesion as suggested by biomimetic model studies. The repellers are linked through FERM binding proteins (say ezrin) to the actin cortex and can actively control the shape of adhering cells, such as the polarization of T-cells in lymph tissue.^8,9^ The front adheres tightly to the APC through integrin–ICAM-1 links (forming immunological synapses or SMACs), while the repellant selectins and CD43 move to the trailing end (uropod), where they can adhere weakly on endothelial cells or tissue through the repellers.

#### Microtubule as the scaffolding complex and cell polarizer

The microtubule (MT) plus-end associates with F-actin *via* plus-tip proteins (Clip 170) and acts as a scaffolding complex which recruits several effector complexes involved in the structuring of the actin network. First, it can recruit Rac-1 and WASP and trigger the local growth of branched Arp2/3 linked actin (Fig. 13d).^82^ Second, it can associate with ezrin and activated Rho-A GTPases to trigger the formation of active micro-muscles that couple to adhesion domains (Fig. 13c), facilitating the retraction of uropods during cell migration.

The global cell shape is stabilized by passive and active coupling of the microtubules to the actin cortex. Active coupling is mediated by the ADAP–dynein complexes (as shown in Fig. 11c), and passive coupling mediated by plus end binding proteins (+TIPs) such as the IQGAP-1/Clip 170 complex (Fig. 13d). The traction force in the first case is determined by the number (*n*) and activity (force *f*) of dynein motors: *Σ*_act_ = *nf* and in the second by one-dimensional analogon of eqn (4a). In equilibrium the sum of all traction forces must be zero:

(10) $$\sum_{1}{\vec{\Sigma }}_{i}\;=\;\sum_{i}{\vec{\Sigma }}_{i,\mathrm{act}}\;+\;\sum_{i}{\vec{l}}_{i}w{\left(1-\cos\;\theta_{ci}\right)}^{-1}$$

Here, *l*_i_ is a unit vector in the direction of the microtubule, and *w* is the energy per unit length gained by the binding of actin to microtubuli through the plus end proteins (Fig. 13b). Any shape change triggered by external forces can be balanced by changes in the traction forces. The adaption of the actin–MT coupling is very rapid (~0.1 s).^74^ The complex between MT tips and ezrin triggers the activation of the Rho-A GTPase. This results in the self-assembly of micromuscles facilitating the retraction of the uropod during cell migration.

### Adhesion controlled pathfinding of axons by filopodia

The growth of axons in tissue is guided by interplay of cell–cell and cell–tissue adhesion. Axon growth cones penetrate in a quasi-random fashion through the tissue. From the tip of the growth cone and the shaft, finger-like extensions (filopodia) protrude randomly, searching for signals from other cells or for adhesion sites. Two major regulators of axon pathfinding are integrin–laminin adhesion domains and signal molecules of the ephrine family recognized by specific cell surface receptors. The physical basis of axon guiding is not understood yet. Two scenarios controlled by adhesion mediated signaling pathways have been identified, the mechanisms of which were suggested previously in our cell-mimetic studies.^87^

#### The laminin mediated pathway

**Fig. 14:**
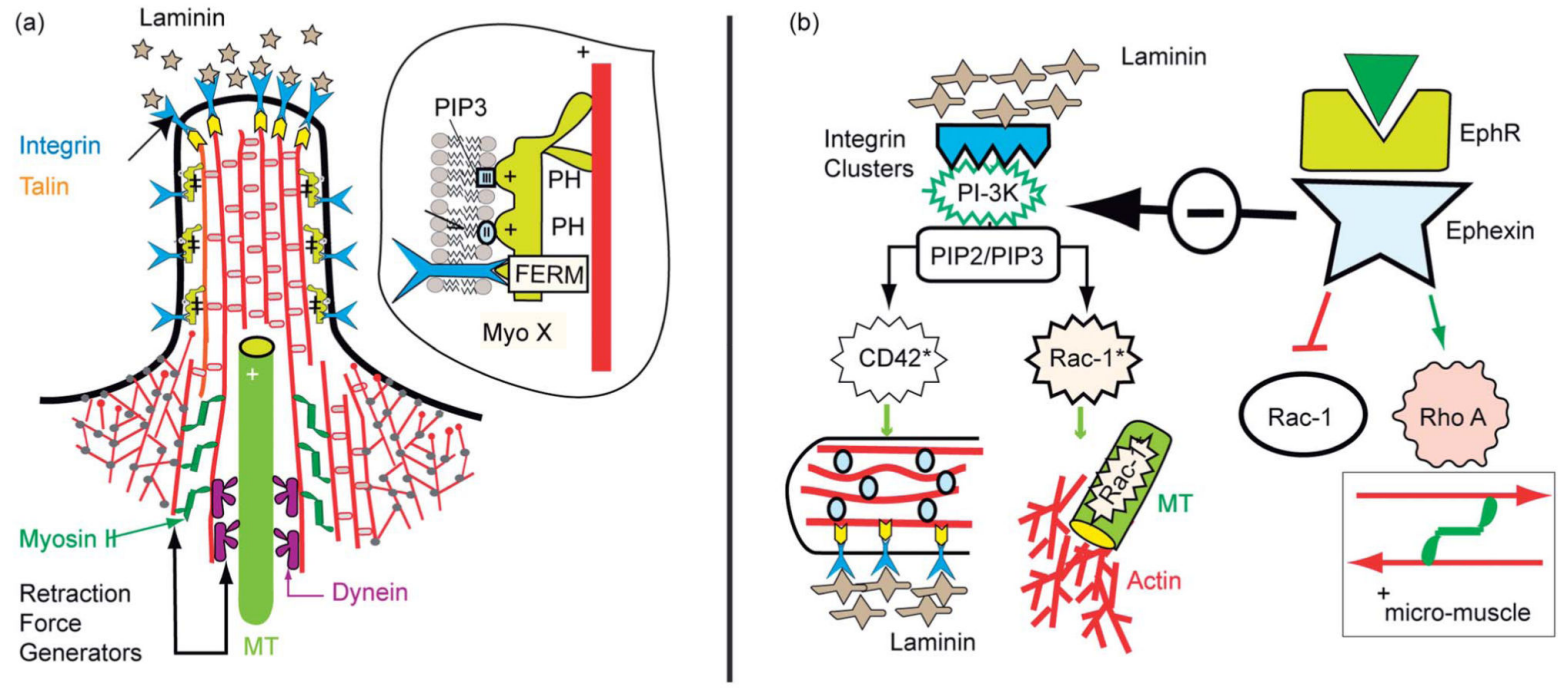
Protrusion and retraction of filopodia. (a) Filopodium with partially penetrating microtubules and assemblies of actin–myosin II micro-muscles at the base of actin bundles. The MT tip can recruit Rac triggering the branched actin gel growth as shown in (b). At the base actin–myosin stress fibers can be formed by activated Rho-A GTPase which facilitate the retraction of the filopodia. The tip can form adhesion domains by binding of integrin to laminin clusters (see text). (b) Left side: activation of GTPase (Rac-1, Cdc42) through PI-3K*, stimulated by integrin–laminin clustering. The activated GTPases are bound to stable MT and to actin bundles via IQGAP/Clip 170 complexes (see GlossarSX). Thus branched and bundled actin gels can form. Right: activation of Rho-A by the ephrin mediated pathway which activates Rho A through the specific GEF ephhexin, but inhibits the PI-3K mediated pathway.

Filopodia are pushed forward by prolongation of actin bundles, triggered by stimulation of the growth promotor formin (Dial-1) activated by the GTPase Cdc42.^83^ The protrusions encountering clusters of laminin in the tissue are stabilized by recruitment of high affinity integrins (α_6_β_1_) and by binding of FERM proteins (such as ezrin), to the integrin β-chains (Fig. 13b). This requires the assembly of a minimum number of integrins and PIP2/PIP3 at the tip of the several ten μm long protrusions. The PIP2/3 facilitates the activation of Cdc42 recruitment to the plasma membrane through electrohydrophobic forces.^84^ Integrins and PIP2/3 can be enriched at the tip by rapid transport through the motor protein myosin X which exposes both FERM domains and PIP2/3-binding pleckstrin homology domains.^85–87^ The stable filopodia act as loci for the growth of the branched Arp2/3-linked actin network by activation of WASP through MT-coupled activated GTP-Rac-1 (left panel in Fig. 14b). Moreover, Rac-1 facilitates the stabilization of the MT by de-activation of the microtubule destabilizing factor stathmin.

#### The ephrin-mediated pathway (Fig. 15)

**Fig. 15:**
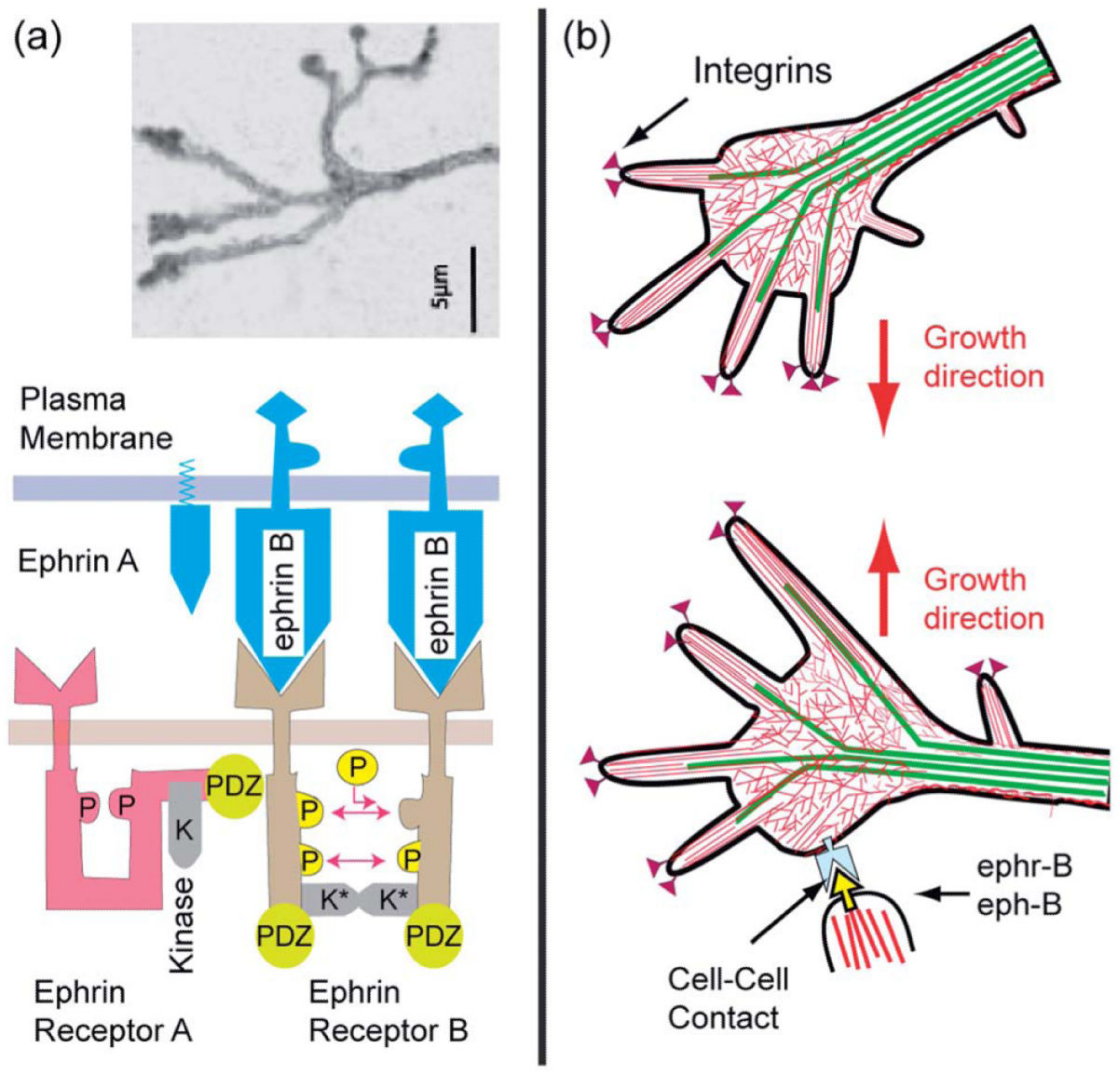
Ephrine mediated retraction of filopodia and redirection of axon growth. (a) Top: typical view of the tip of axon growing on laminin covered surfaces (modified after ref. 89). Bottom: schematic view of membrane bound signal molecules ephrins (A and B) and receptors ephr-B (of RTK type) embedded in the top and bottom membrane, respectively. The left side shows a single receptor in the sleeping and the right a dimer in the active conformation, in which the tails are mutually phosphorylated and the kinase K is activated. Note that the stimulation of the ephr-B occurs by mutual phosphorylation of the cytoplasmic domains of two receptors. Activation is therefore only triggered by clusters of eph-R (b) retraction of filopodia at bottom by activation of Rho-A GTPase through the interaction of the ligand eph-B, exposed by target neurons, with receptor ephr-B on a growing axon. The axon grows in a new direction.

The long range pathfinding of axons is controlled by signaling molecules of the ephrin (eph) family and the conjugate receptors (ephR). Ephrins are membrane bound and require cell–cell contact to become active. Fig. 15 shows the repulsion of axon cones by neurons exposing ephrine B2 binding to ephr-B.^88^ This triggers the activation of Rho-A *via* the specific guanine exchange factor (GEF) ephexin which is activated by membrane anchoring through binding to PIP2/PIP3 *via* pleckstrin domains. Excited GTP-Rho-A activates the kinase ROCK which switches on the myosin light chain kinase (MLCK) and triggers the activation of actin–myosin micro-muscles in the growth cone (as shown on the right side of Fig. 14b).^89^ They can be pulled back by the filopodia, provided they are not bound strongly to the substrate *via* laminin–integrin clusters. To facilitate the retraction of filopodia, the adhesion strength can be reduced by two universal mechanisms. First, by switching the integrins from the high to the low affinity state through deactivation of the GTPase R-Ras, the major activator of the high affinity state through activated ephr-B*, second, by decomposing talin (or ezrin) through the protease calpain. This enzyme is activated by binding of R-Ras to the transmembrane protein Fram 38, which triggers the release of Ca and activates the protease.^90^

## Conclusions and perspectives

Cell adhesion is a field of life science that implies rich physics. Over the last 10 years systematic studies of the adhesion of giant vesicles on supported membranes containing the major ingredients mediating cell adhesion have provided valuable insights into the physical basis of cell adhesion. They stimulated the development of sophisticated theoretical models of cell adhesion and experimental tools enabling local measurements of adhesion forces.

Giant vesicles containing small amounts of attractive CAMs mediated the formation of strong specific inter-membrane links with co-receptors of target membranes. In the presence of moderate concentrations of repeller molecules, mimicking the glycocalyx of cells, vesicles adhered by formation of microdomains of tight adhesion, which are separated by regions of a membrane residing in the minimum of the nonspecific potential (see Fig. 2). The adhesion strength of the domains is sensitively controlled through the 2D osmotic pressure of the non-committed attractive CAMs and repellers. In cells the adhesion domains also form by lateral phase separation within the lipid protein bilayer. They are, however, enforced in a second process by coupling of the actin gel patches to the intracellular domains of clustered CAM–CAM pairs which links adhesion processes to cell signaling pathways. The adhesion of both vesicles and cells is further controlled by entropic repulsion forces generated by pronounced bending excitations. They counteract the van der Waals attraction, drive the lateral attraction of CAMs and the formation of adhesion domains by pushing the glycocalyx aside.

The model studies revealed a fundamental difference between cell–cell adhesion with the receptors on both surfaces mobile and cell–tissue interactions where the co-receptors are immobilized which becomes evident under external forces. In the latter case bonds break at the front and reform closer to the center but eventually the adhering shells unbind. In the former case the adhesion domains are enforced by condensation of the links and by increasing the binding strength. Such rapid adhesion strength enforcements by force induced linker condensation play a key role in the sub-second adaption of cell–cell adhesion strength under varying hydrodynamic shear forces. This generic adaption provides the cells enough time to adapt the adhesion strength by modulation of the actin–receptor coupling through cell signaling.^78^ The adhesion domains provide numerous biological advantages. They enable migrating cells to rapidly form transient adhesion domains at the leading edge (to transmit momentum to the substrate) and dismantle mature ones to retract the trailing end without loss of material. The adhesion strength can be adapted to external force fluctuations within seconds by varying the density of attractive and repellant CAMs through exocytosis and endocytosis or by protease-mediated dismantling of talin mediating the CAM–cytoskeleton links.

Adhesion domains play a key role as local biochemical reaction centers that control the access of activators and/or inhibitors as shown for the stimulation of T-cell proliferation by active immune synapses. They simultaneously serve as integrative platforms for the parallel switching of two genetic expression pathways required for T-cell stimulations (see Fig. 9).

The polarization of migrating cells is mediated by global reorganization of attractive and repellant CAMs, whereby the cell shapes are stabilized by passive and active binding of MT plus ends to adhesion domains. In addition, the MT plus ends form scaffolding complexes that trigger the recruitment of activators of actin growth, such as GTPases acting as molecular switches which activate the actin cross-linkers generating actin bundles (such as formins) or branched actin gels (such as Arp2/3).

The global organization of the adhesion domains formed by different specific CAM–CAM pairs and the polarized cell shape is regulated by coupling of the intracellular domains of attractive and repellant CAMs to the actin cortex followed by actin–microtubule crosstalk. The actin–membrane coupling is mediated by linker proteins exposing a specific class of protein homology domains, called FERM domains. An important member is talin that couples actin to the beta chains of integrins and simultaneously triggers the transformation of these CAMs from a low to a high affinity state (see Fig. 6). In the cell resting states the actin membrane linkers reside in the cytoplasm in a sleeping conformation. They are activated by adsorption to clusters of negatively charged lipids (such as phosphoinositides) through electrostatic–hydrophobic forces and thus provide a major link between cell adhesion and cell signaling.

As a final example, we discuss the search for target cells migrating in tissues by slender axon cones generating filopodia. We show how they are guided by attractive and repellant forces arising between the axon tips and target tissues or cells, respectively. The attractive pathway is mediated by adhesion domains, the formation of which is triggered by the extracellular protein laminin mediating integrin clustering, which attract microtubule plus ends. The MT plus ends act as scaffolding complexes and reaction platforms for actin gel formation and the formation of a new axon growth cone. Simultaneously, the GTPase switches that activate the actin growth promoters suppress the activity of the MT destabilizing proteins such as stathmin. The repulsion of filopodia is mediated by adhesion domains formed by links between the membrane bound signaling molecule ephrin-A and the conjugate ephrin receptor-B, residing in the target membrane and the filopodia tip, respectively. They trigger simultaneously the activation of actin myosin micro-muscles *via* Rho GTPases and the weakening of the adhesion domain strength by talin dismantling, resulting in the retraction of the exploring protrusions and the changing of the axon growth direction.

### Future challenges

Adhesion is a paradigm of the ubiquitous interplay of cell signaling, the modulation of material properties and the biological functions of cells. The function of interactive biochemical and genetic networks is currently actively studied by bio-informaticians providing insights into the interactive control of cell signalling pathways. However, a full understanding of cell adhesion requires insights into the changes of the composition and physical properties of the composite cell envelopes by biochemical signaling pathways and genetic expression. To gain deeper insight into these aspects of cell adhesion a future challenge of biomimetic studies is the design of more realistic cell models by reconstitution of actin cytoskeleton into giant vesicles doped with reconstituted CAMs, such as integrin.^91^ In this way the regulation of vesicle adhesion by actin membrane crosstalk could be studied.

## Figures and Tables

**Fig. 10:**
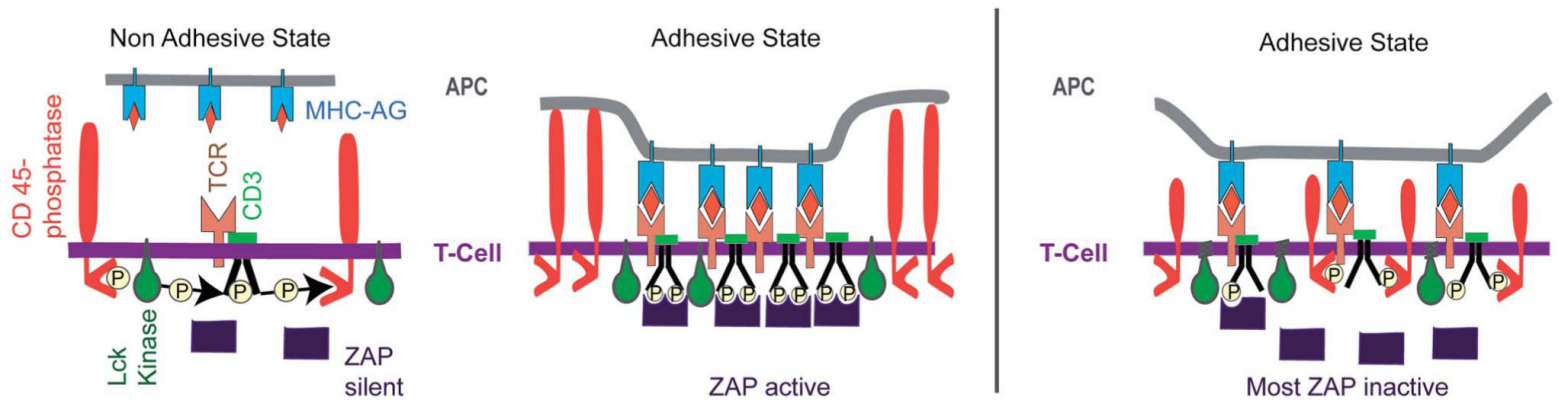
Left panel: model of activation of T-cells by adhesion domains formed during the initial phase of T-cell–APC encounters (before the formation of large central SMACs). The kinase ZAP-70 is activated by binding to the phosphorylated tyrosine groups of the cytoplasmic chain of the co-receptor CD3. The non-adhesive state occurs due to the abolishment of ZAP activation by CD45-mediated ongoing de-phosphorylation of CD3. The adhesive state leading to the formation of immune synapse (IS) is promoted by the clustering of bound TCR–MHC–AG pairs, which results in the expulsion of the inhibitor CD45 from the reaction center by steric forces. Right panel: demonstration of the CD45 self-inhibition model by Choudhuri and collaborators.^64^ By reducing the length of the extracellular domain, CD45 can diffuse into the tight adhesion domain and prevent the activation of ZAP by continuous removal of phosphate groups at the CD3-coreceptor. The same effect was observed by prolongation of the extracellular domains of the MHC–receptor. In summary, the phosphatase CD45 plays a twofold role: it inhibits the CD3-phosphorylation, and, together with other glycoproteins of the glycocalyx (e.g. CD43), acts as a buffer molecule counteracting adhesion.

## Footnotes

† Repeller molecules: in the present nomenclature repellers or repellant CAMs are cell surface proteins with long extracellular tails or high negative charges which generate generic repulsion forces and impede short range attractions.
